# Tissue and plasma proteomic profiling indicates AHSG as a potential biomarker for ascending thoracic aortic aneurysms

**DOI:** 10.1186/s12872-023-03154-6

**Published:** 2023-03-16

**Authors:** Rafailia Kazamia, Anna Keravnou, Areti Moushi, Kleitos Sokratous, Kyriaki Michailidou, Kristia Yiangou, Marinos Soteriou, Stavroulla Xenophontos, Marios A. Cariolou, Evy Bashiardes

**Affiliations:** 1 Department of Cardiovascular Genetics and The Laboratory of Forensic Genetics, The Cyprus Institute of Neurology and Genetics, Iroon Avenue 6, Agios Dometios, 2371 Nicosia, Cyprus; 2 OMass Therapeutics, The Schrödinger Building, Heatley Road, The Oxford Science Park, Oxford, OX4 4GE UK; 3 Biostatistics Unit, The Cyprus Institute of Neurology and Genetics, Iroon Avenue 6, Agios Dometios, 2371 Nicosia, Cyprus; 4 Department of Cancer Genetics, Therapeutics and Ultrastructural Pathology, The Cyprus Institute of Neurology and Genetics, Iroon Avenue 6, Agios Dometios, 2371 Nicosia, Cyprus; 5 Department of Cardiology and Cardiovascular Surgery, American Medical Centre, Spyrou Kyprianou Avenue 215, 2047 Nicosia, Strovolos Cyprus

**Keywords:** Thoracic aortic aneurysm, TAA, Biomarkers, Proteomic, Mass spectrometry, Aortic tissue, Plasma, AHSG, Blood-borne

## Abstract

**Background:**

Thoracic Aortic Aneurysms (TAAs) develop asymptomatically and are characterized by dilatation of the aorta. This is considered a life-threatening vascular disorder due to the risk of aortic dissection and rupture. There is an urgent need to identify blood-borne biomarkers for the early detection of TAA. The goal of the present study was to identify potential protein biomarkers associated with TAAs, using proteomic analysis of aortic tissue and plasma samples.

**Methods:**

Extracted proteins from 14 aneurysmal and 12 non-aneurysmal thoracic aortic tissue specimens as well as plasma samples from six TAA patients collected pre-and postoperatively and six healthy controls (HC), were analyzed by liquid chromatography-tandem mass spectrometry. Proteomic data were further processed and following filtering criteria, one protein was selected for verification and validation in a larger cohort of patients and controls using a targeted quantitative proteomic approach and enzyme-linked immunosorbent assay, respectively.

**Results:**

A total of 1593 and 363 differentially expressed proteins were identified in tissue and plasma samples, respectively. Pathway enrichment analysis on the differentially expressed proteins revealed a number of dysregulated molecular pathways that might be implicated in aneurysm pathology including complement and coagulation cascades, focal adhesion, and extracellular matrix receptor interaction pathways. Alpha-2-HS glycoprotein (AHSG) was selected for further verification in 36 TAA and 21 HC plasma samples using targeted quantitative proteomic approach. The results showed a significantly decreased concentration of AHSG (*p* = 0.0002) in the preoperative plasma samples compared with HC samples. Further analyses using a larger validation dataset revealed that AHSG protein levels were significantly lower (*p* = 0.03) compared with HC. Logistic regression analysis on the validation dataset revealed males, advanced age, hypertension and hyperlipidaemia as significant risk factors for TAA.

**Conclusion:**

AHSG concentrations distinguish plasma samples derived from TAA patients and controls. The findings of this study suggest that AHSG may be a potential biomarker for TAA that could lead to better diagnostic capabilities.

**Supplementary Information:**

The online version contains supplementary material available at 10.1186/s12872-023-03154-6.

## Background

Thoracic aortic aneurysm and dissection (TAA/D) is a life-threatening condition which is ranked in the top 20 leading causes of death worldwide. It is difficult to determine the exact incidence due to cases of sudden death out of hospitals and misdiagnosis [1]. A pooled incidence calculated based on 22 population studies represented an overall incidence of 5.3 per 100, 000 individuals per year [2]. TAAs can be classified as syndromic, non-syndromic familial or sporadic. The natural history of the disease remains elusive and not well understood [3]. TAA/D is caused by weakness in the arterial wall due to apoptosis of smooth muscle cells (SMCs) and degeneration of the aortic media. Cigarette smoking, male sex, bicuspid aortic valve, atherosclerotic disease, high blood pressure, positive family history and/or genetic predisposition to the disease are established risk factors for TAA/D [4, 5]. Although this aortic disease affects males more often than females, the latter are characterized by poorer outcomes and higher mortality rates [6].

The normal diameter of the thoracic aorta is approximately 30 mm and it is considered aneurysmal when it exceeds 50 mm or increases to one and a half times more than the aorta’s normal width. The greater the diameter of the aortic wall, the higher the probability of aortic dissection and rupture, leading to high mortality [1]. TAA/D has a high in-hospital mortality rate of 57% without emergency surgery and 17% to 25% with emergency surgery in accordance with national and international registries [7, 8]. Therefore, early diagnosis and surgical repair are critical for the survival of patients [9]. Aorta replacement surgery is based on aortic diameter and the medical history of the patient. For all symptomatic (rupture, dissection, or with pain) TAA patients it is recommended to undergo vascular repair while this is not recommended for the asymptomatic patients until the risk of rupture or dissection exceeds the risk associated with surgery [1, 9]. Elective vascular repair of ascending TAAs is recommended for aortic diameter > 55 mm or for patients with rapid expansion > 5 mm per year, progressive aortic incompetence or familial cases with dissection or rupture to avoid aortic dissection or rupture in smaller aortic wall sizes [10]. The aforementioned aortic wall sizes are not an absolute measure and each patient’s assessment should be individualized based on additional factors such as body size, general comorbidities and aneurysm growth [1, 9].

In more than 95% of cases, TAA/D remains asymptomatic and it is identified incidentally when a patient undergoes imaging, such as echocardiography, computed tomography scanning, or magnetic resonance imaging, for other reasons [3, 11]. Therefore, there is an urgent need for highly sensitive and specific blood-borne biomarkers that can detect TAAs in asymptomatic patients, proactively [12]. Plasma represents the largest and deepest version of the human proteome that is present in any sample [13], offering the most suitable biological fluid for isolating candidate protein markers.

Identification of circulating biomarkers with traditional hypothesis-based studies has so far been unsuccessful in identifying a clinically applicable biomarker for TAAs. Their focus was on the protein components of the arterial wall, such as the different collagen types, elastin, tropoelastin, fibulin, and matrix metalloproteinases (MMPs), all playing a significant role in the strength as well as the flexibility of the aorta [14, 15]. Advances in mass spectrometry (MS)-based technology have permitted the non-hypothesis-based proteomic approaches to be pursued attempting to discover potential candidate markers for TAAs [16, 17]. Currently, no biomarker implicated in early detection and screening of TAAs is available.

The aim of the present study was to identify potential plasma-based protein biomarkers related to TAAs. To achieve this, thoracic aneurysmal and non-aneurysmal aortic tissue specimens and plasma samples from TAA patients and controls were collected. Untargeted MS-based proteomic analysis on both, tissue and plasma samples, led to the identification of several differentially expressed proteins (DEPs). One protein was selected for further investigation after applying strict filtering criteria and its relative concentration level was measured by liquid chromatography-multiple reaction monitoring-mass spectrometry (LC-MRM-MS) technique and enzyme-linked immunosorbent assay (ELISA).

## Methods

### Sample collection

The present study received approval from the Cyprus National Bioethics Committee (Project Approval ID: ΕΕΒΚ/ΕΠ/2014/32). All subjects signed informed consent forms before participating. Patient recruitment for this project was an ongoing process. Therefore, throughout the stages of the experimental work, the number of samples included differs. The baseline demographic characteristics of the study participants included in the discovery phase can be found in Table 1. The patients with known aortic aneurysm cases diagnosed before surgery were on beta blockers (80%). The emergency cases, were not treated with betablockers before surgery.

**Table 1:** Baseline demographic characteristics of individuals included in the discovery phase

	**Discovery Phase (1) LC–MS-MS**					
Sample type	Tissue samples			Plasma samples		
Study groups	CABG	TAA	*p*	Control	TAA	*p*
Sample number	*n* = 12	*n* = 14	NA	*n* = 6	*n* = 6	NA
Age: mean (SD)	65.2 (12.8)	64.9 (12.3)	0.9	37.8 (9.8)	67 (13.7)	0.002
Male/Female	12/0	14/0	NA	4/2	4/2	1
Hypertension, n (%)	NA	11 (79%)	NA	0 (0%)	5 (83%)	NA
Diabetes, n (%)	NA	0 (0%)	NA	0 (0%)	0 (0%)	NA
Hyperlipidaemia, n (%)	NA	3 (25%)	NA	0 (0%)	0 (0%)	NA

*LC–MS-MS* liquid chromatography-tandem mass spectrometry, *CABG* coronary artery bypass graft, *TAA* thoracic aortic aneurysm, *SD* standard deviation, *NA* not available

The baseline characteristics of the study participants included in the verification and validation phases can be found in Table 2.

**Table 2:** Baseline demographic characteristics of individuals included in the verification and validation phases

	**Verification Phase (2): LC-MRM-MS**			**Validation Phase (3): ELISA**		
Sample type	Plasma samples			Plasma samples		
Study groups	Control	TAA	*p*	Control	TAA	*p*
Sample number	*n* = 21	*n* = 18	NA	*n* = 56	*n* = 48	NA
Age: mean (SD)	57.1 (3.6)	66 (8)	0.0001	60.61 (8.2)	64.88 (9.4)	0.008
Male/Female	9/12	12/6	0.1	30/26	36/12	0.02
Hypertension, n (%)	0 (0%)	13 (72.2%)	0.000002	13 (23.2)	37 (77.1)	< 0.0001
Diabetes, n (%)	0 (0%)	2 (11%)	0.1	6 (10.7)	5 (10.4)	0.1
Hyperlipidaemia, n (%)	0 (0%)	10 (55.6%)	0.00007	13 (24.1)	25(52.1)	0.002

*LC-MRM-MS* liquid chromatography-multiple reaction monitoring-mass spectrometry, *ELISA* enzyme-linked immunosorbent assay, *TAA* thoracic aortic aneurysm, *SD* standard deviation, *NA* not available

### Aortic tissue samples

Thoracic aneurysmal tissue samples (T) were collected from 14 male patients with sporadic ascending TAAs undergoing surgery for the repair of the aneurysm at the American Medical Centre (AMC), Cyprus. The criteria used for the selection of patient samples during each phase were, that all patients presented with an aortic diameter ≥ 50 mm and had no transfusions during or immediately after the surgery. Upon initial evaluation, patients had no manifestation of phenotypic findings hinting toward connective tissue disorders or aortic pathology. More specifically, the exclusion criteria for these TAA patients were as follows: (1) patients with a known family history of TAA, (2) patients diagnosed with known genetic conditions related to aneurysm formation, such as Marfan syndrome, Ehlers-Danlos syndrome or congenital disorders, (3) patients diagnosed with aneurysm in parts of aorta other than in the ascending thoracic segment, and (4) patients with a bicuspid aortic valve. During the repair procedure, the ascending sort extending from the sinotubular junction to the proximal aortic arch was resected. The resected aorta was replaced with a synthetic graft. The T samples were taken from the widest region of the ascending aorta at the time of surgery and then rinsed in saline solution, cut into sections and each section placed in a separate tube, snap-frozen in liquid nitrogen and stored at − 80 °C.

Aortic tissue punches from patients who underwent coronary artery bypass graft (CABG) surgery were also collected and served as control aortic tissue (CT) samples which were the closest possible to non-affected or normal aorta. Specifically, one to three punches of 3-4 mm in diameter generated during the normal operating procedure were derived from the normal (non-affected) part of the aorta. The excised samples were rinsed in saline solution, snap- frozen in liquid nitrogen and stored at − 80 °C.

### Plasma samples

Blood samples were collected from TAA patients at different time points. A small blood sample (~ 2 ml) was collected from the patients pre-operatively (Blood Before, BB) and a second sample at 8–10 days post-operatively (Blood After, BA). The collection of the BA samples was performed prior to the discharge of the patients from the AMC since most patients did not return to the AMC after surgery for follow-up, and instead visit their personal cardiologist for further medical follow-ups. Wherever possible, a third blood sample was collected, one-month post-operatively (Blood After Month, BAM) in cases where the patients visited the AMC for their medical monitoring. Thus, BAM samples represent a more comparable near-normal-state condition, post- operatively rather than the BA samples. The blood samples were collected in BD Vacutainer blood collection tubes with EDTA and precautions were taken to reduce the possibility of haemolysis. When the blood samples were collected, the needle was removed from the syringe and the blood slowly expelled into the tube. The plasma was isolated within 30 min after collection, in a cooled centrifuge using the double centrifugation protocol which included a first centrifugation spin at 3000 rpm for 10 min, followed by a second spin of the plasma sample at 3200 rpm for 10 min to remove residual cells. Additional blood samples were also collected from individuals who declared themselves free of any symptoms of cardiovascular disease (CVD), comprising the healthy control (HC) population and were used in the three experimental phases.

### Study design

The study was divided into three phases including, the discovery phase using untargeted MS-based proteomic analysis of aortic tissue and plasma samples, the verification phase for the quantification of the selected protein in plasma samples using LC-MRM-MS and finally, the validation phase on a larger number of plasma samples using ELISA.

**Fig. 1:**
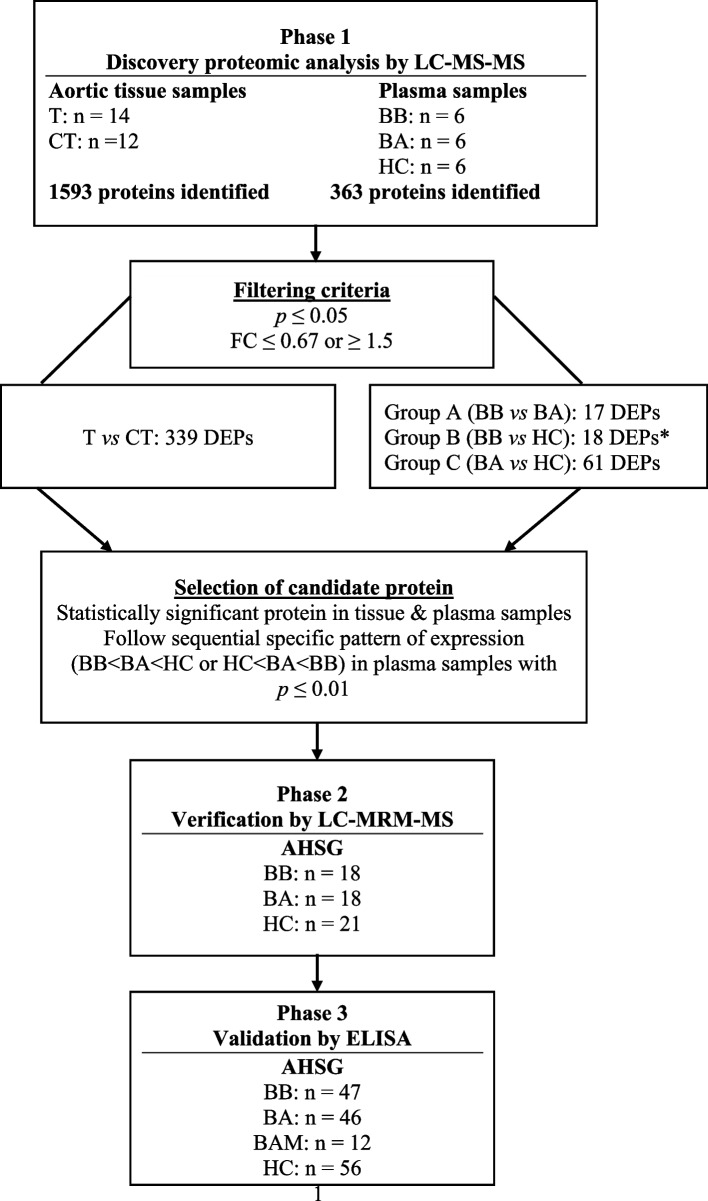
Workflow of the study design. Phase 1: Discovery proteomic analysis for the identification of DEPs. Phase 2: Verification of Alpha-2-HS glycoprotein (AHSG). Phase 3: Validation of AHSG protein. LC–MS-MS, liquid chromatography-tandem mass spectrometry; T, thoracic aneurysmal tissue sample; CT, control aortic tissue punches; BB, plasma from blood sample collected pre-operatively; BA, plasma from blood sample collected 8–10 post-operatively; BAM, plasma from blood sample collected one-month post-operatively; HC, healthy control; DEPs, differentially expressed proteins; FC, fold change; LC-MRM-MS, liquid chromatography-multiple reaction monitoring-mass spectrometry; ELISA, enzyme-linked immunosorbent assay; AHSG, Alpha-2-HS-glycoprotein. * indicates the sample group comparison from which the candidate protein was selected

In the discovery phase both tissue and plasma samples were interrogated because we expected a correlation between the two samples since all tissue proteins leak into the blood as a result of tissue damage (leakage markers). Therefore, any potential protein markers selected for further analysis in our plasma samples would have originated from the aneurysmal tissue [13, 18]. A workflow summarizing the three phases of the study is shown in Fig. 1.

### Sample preparation for MS-based analyses

#### Protein extraction from aortic tissue samples (T & CT)

Each aortic tissue sample (approximately 1 cm x 1 cm square) was solubilized in lysis buffer (10 mM Tris–HCl pH 7.4, 150 mM NaCl, 1 mM EDTA, PBS), containing protease inhibitors (cOmplete Mini EDTA-free, Roche, Germany). After, overnight incubation with acetone at -20 °C, the sample was centrifuged (5 min, 1000 × g), the acetone was discarded and the precipitated protein pellet was air-dried and stored at -20 °C, until further processing.

#### Immunodepletion of plasma samples

Immunodepletion of abundant plasma proteins during plasma discovery phase was performed using the ProteoPrep® Immunoaffinity Albumin & IgG Depletion Kit (Sigma-Aldrich, USA), which was employed for the removal of albumin and IgG, according to the manufacturer’s protocol. For the targeted LC-MRM-MS, the Human 14 Multiple Affinity Removal Spin Cartridge, MARS 14 (Agilent Technologies, 5188–6560, USA) was employed for the removal of 14 high abundance proteins, following the manufacturer’s instructions. Removal of abundant proteins from plasma samples was followed by protein extraction by acetone precipitation as described above.

#### Trypsin digestion and protein purification

Filter aided sample preparation (FASP) was used to prepare the samples for MS analysis following a modified protocol [19]. Briefly, protein pellet from either tissue or plasma samples was dissolved in urea (8 M), the proteins were reduced by dithiothreitol, alkylated by iodoacetamide and then digested by trypsin at a ratio of 1:50 (trypsin: protein) for 18 h at 37 °C. In case of targeted LC-MRM-MS, synthesized isotopically labelled peptide (PepScan, Lelystad, The Netherlands), was spiked-in to each plasma sample after trypsin digestion and used as stable internal standards (SIL) for the quantification of the targeted protein and the monitoring of the immunodepleted proteins. The resulting tryptic peptides were purified and desalted using C18 solid phase extraction cartridges (Sep-Pak tC18, Waters, Austria), and the peptides were dried in a vacuum centrifuge and stored at -20 °C until further processing.

#### Untargeted MS-based proteomic analysis

Purified peptides were re-suspended in an aqueous buffer (99% H_2_O, 1% acetonitrile) and adjusted to a final concentration of 0.2 μg/μL. Peptides were separated on a nanoAcquity UPLC system (Waters, UK) with an Acquity UPLC M-Class peptide BEH C18 column (Waters, 75 μm × 150 mm, 1.7 μm, 300) and analyzed on a Synapt G2Si MS instrument (Waters, UK), operated on ion mobility mode using the UDMSe method [20]. Analysis of the MS data was carried out by Progenesis QI for proteomics software (QIp) against the UniProtKB/SwissProt human reference proteome database (https://www.uniprot.org/proteomes/UP000005640) [21].

#### Selection of candidate biomarkers

The expression pattern of proteins was assessed in the tissue and plasma samples as the aim was to identify circulating TAA biomarkers. In order to select candidate proteins for further verification and validation, emphasis was given to the significantly DEPs identified in the discovery phase by comparing BB *vs* HC plasma samples. Additionally, the filtered proteins from the three group comparisons were further examined to identify whether any of them follow a sequential specific pattern of expression such as lower protein expression (downregulated with FC ≤ 0.67) in BB samples compared to HC samples (upregulated with FC ≥ 1.5) or vice versa. Thus, further stringent selection criteria were applied to limit the number of candidate proteins that will be incorporated in the verification phase. More specifically, we selected proteins that followed the aforementioned sequential pattern of expression with *p* ≤ 0.01 among BB *vs* HC, suggesting that the candidate proteins were blood-borne and TAA-specific, as a result, one protein remained for further verification using LC-MRM-MS.

#### Verification of AHSG protein

Peptides were reconstituted in an aqueous buffer (99% H_2_O, 1% acetonitrile and 0.1% formic acid). The sample’s total protein concentration was measured on a Nanodrop ND-1000 Spectrophotometer (Thermo Fisher Scientific, UK) and the sample was used without any further dilution. To account for protein concentration variance due to sample preparation, the values from LC-MRM-MS analysis for each sample, were normalized by multiplying them by a correction factor that was calculated based on the total protein concentration measured in each sample (see Additional file 1-Table S1.1). Peptides were separated on an Acquity I-Class UPLC system (Waters, UK) using a C18 column (1 mm × 150 mm, 1.7 μm, Waters, UK) and analyzed on a Waters Xevo TQD MS system operated on MRM mode. The absolute quantification of the selected protein in plasma samples was performed using SIL peptide as internal standard. The peptide sequence of the targeted protein, AHSG was FSVVYAK. The MRM transitions and instrument parameters for each peptide (targeted and immunodepleted peptides) are shown in Additional file 2-Table S2.1. The targeted proteomic data were interpreted using the TargetLynx™ and Skyline software (version 4.2).

#### Validation of AHSG protein

The concentration of AHSG was determined in plasma samples using the commercially available ELISA kit [AHSG (#: SK00173-20) [Aviscera Bioscience, Inc, Santa Clara, USA]. The assay was performed according to the manufacturer’s instructions. The dilution series for the standard curve preparation were prepared using the stock solution that serves as the high standard, supplied with the respective kit. All samples, standards and controls were analyzed in duplicate and measured at 450 nm using the iEMS Reader MF (Labsystems, Helsinki, Finland). The optical density data were imported and analyzed by MyAssays Ltd (https://www.myassays.com/) “Four Parameter Logistic Curve” online data analysis tool, MyAssays Ltd., 30th June 2021, https://www.myassays.com/four-parameter-logistic.assay [22].

### Statistical analysis

The protein identification and quantification were performed using a less than 1% peptide false discovery rate (FDR). The identified proteins were further filtered using the following criteria: confidence score ≥ 5, sequence length ≥ 6 and hits ≥ 2. Protein-level relative quantitation was performed using the Hi-N approach (N = 3) as implemented in the Progenesis QIp. Volcano plots were constructed using the R statistics software version R 3.6.3 [23], where the y-axis depicts the –log10 (p value) for all the identified proteins and the x-axis shows their respective log2 (Fold Change) which is calculated when comparing diseased versus control samples. Principal components analysis (PCA) were calculated and plotted using the R statistics software version R 3.6.3 [23].

The statistical comparisons in the discovery proteomic phase were carried out using one-way of variance analysis (ANOVA) or Student’s t test, where appropriate. In the verification and validation phases, the data were presented as mean with ± standard deviation (SD). The Shapiro–Wilk’s and Levene’s tests were used to test for normality and equality of variances calculated for two or more groups, respectively. For normally distributed data, unpaired sample two tailed t-test and paired t-test was performed. Wilcoxon Rank Sum and Wilcoxon Signed-Rank Tests were used for non-parametric and paired pairwise comparisons, respectively. Additionally, receiver operating characteristic (ROC) curves were constructed, after the completion of verification and validation phases to assess the discriminatory ability of the selected protein using the GraphPad Prism (version 9). Logistic regression analysis was performed to evaluate the association of risk factors with TAA. In the validation phase, conditional logistic regression was performed adjusted by age in order to explore the effect of age on protein levels. Differences with a p ≤ 0.05 were considered as statistically significant. All statistical analyses were performed using the R (version 4.1.3) and IBM SPSS Statistics for Windows (version 25.0). The statistically significant proteins detected from both aortic tissue and plasma samples were subjected to pathway enrichment analysis using Enrichr (https://maayanlab.cloud/Enrichr/) [24]. Further analysis using STRING (Search Tool for Recurring Instances of Neighbouring Genes) on the DEPs from each discovery phase was performed [25].

## Results

### Phase 1 – Discovery proteomic analysis on aortic tissue samples

**Fig. 2:**
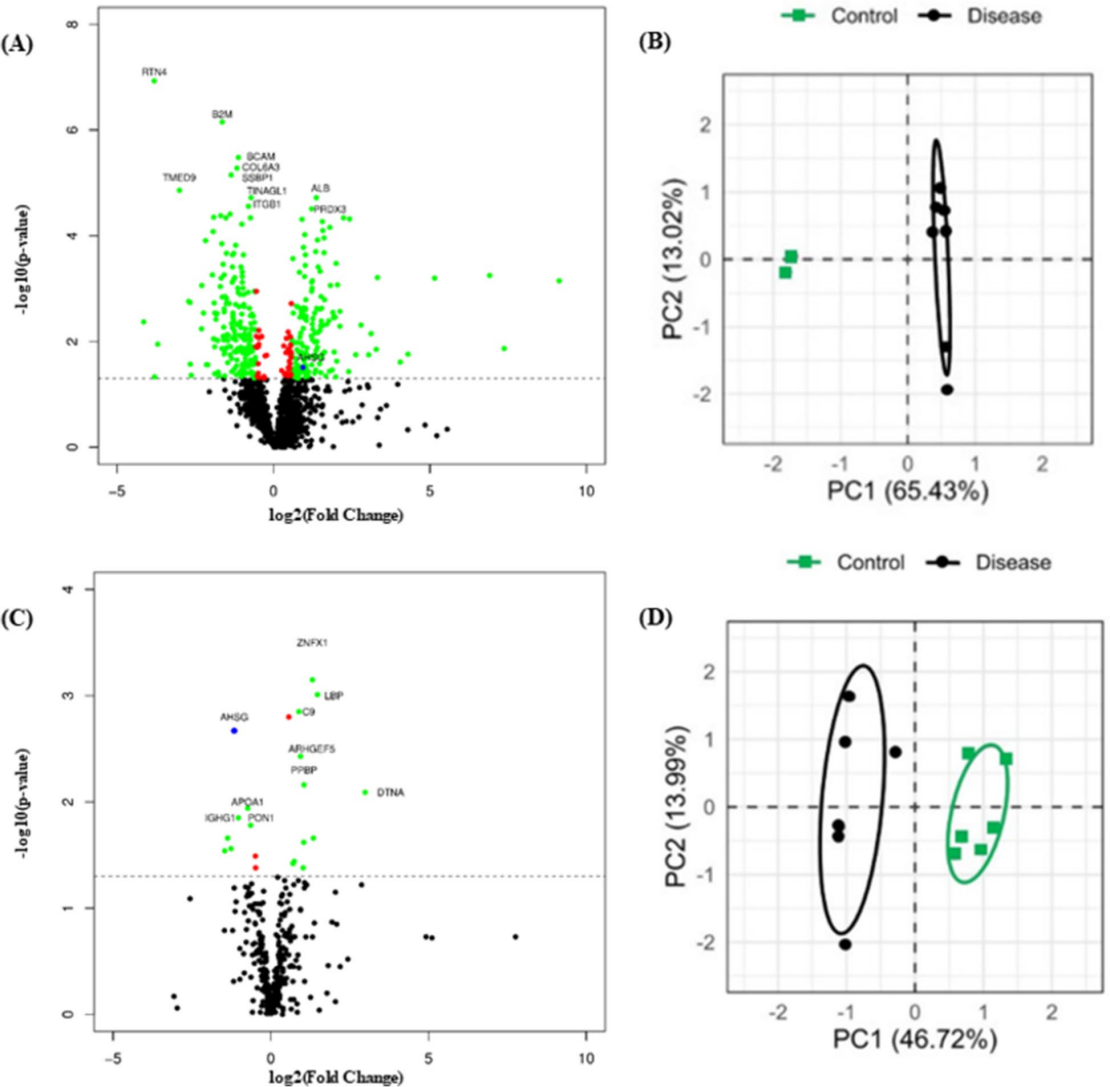
Overview of the distribution of all proteins identified by two discovery proteomic phases. **A** Volcano plot of all proteins quantified in discovery phase of aortic tissue (14 T and 12 CT samples). The x-axis depicts the log2 fold change and the y-axis depicts the –log10 (*p* value) of the discovered proteins in each group comparison. **B** PCA score plot of protein expression pattern of significant proteins shows a clear separation into two distinct clusters of T (black dots) compared with CT (green squares). **C** Volcano plot of all proteins quantified in discovery phase of plasma samples (6 BB and 6 HC). **D** PCA score plots of protein expression pattern of significant proteins shows a clear separation into two distinct clusters of TAA—BB samples (black dots) and HC (green squares). For the volcano plots, the top proteins with the most significant *p* values are labelled with their protein name. Red dots represent proteins with differential expression, *p* ≤ 0.05. Green dots indicate the statistically significant proteins with FC ≤ 0.67 or ≥ 1.5, characterized as down or upregulated proteins, respectively. Black dots indicate the non-statistically significant proteins, *p* > 0.05. The blue dot represents the selected protein. TAA, thoracic aortic aneurysm; FC, fold change; PC, principal component; PCA, principal component analysis; T, thoracic aneurysmal tissue sample; CT, control aortic tissue punches; BB, plasma from blood sample collected pre-operatively; HC, healthy control

The proteomic analysis on 14 T and 12 CT samples was performed to identify DEPs. Overall, a total of 1593 unique proteins were detected and filtered (see Additional file 3; Table S3.1). After filtering, 339 proteins were found to be significantly differentially expressed (*p* ≤ 0.05) from which 185 were downregulated and 154 were upregulated (see Additional file 3-Table S3.2). The volcano plot (Fig. 2A) represents the distribution of the *p* values and the FC from all identified proteins obtained during the discovery analysis. The PCA (Fig. 2B) revealed a clear separation into two distinct clusters between the T and the CT samples.

### Phase 1 – Discovery proteomic analysis on plasma samples

This analysis was performed on 12 TAA plasma samples (six BB and six BA) and six HCs. The goal was to identify DEPs between the three different sample types (BB, BA and HC). The DEPs detected amongst BB *vs* BA, BB *vs* HC and BA *vs* HC were arranged into Group A, Group B and Group C, respectively. Overall, a total of 363 proteins were common between the three groups (see Additional file 3-Table S3.3-S3.5). Finally, 17, 18 and 61 proteins were significantly different in Group A, Group B and Group C, respectively (see Additional file 3-Table S3.6-S3.8). The volcano plot seen in Fig. 2C represents the distribution of the p values and the FC from all identified proteins obtained by comparing BB *vs* HC, which constitutes the samples derived from the affected and control individuals, respectively. The PCA plot shows a clear separation between the BB (black dots) and HC (green squares) samples into two principal components/clusters (Fig. 2D).

### Pathway enrichment analysis

**Fig. 3:**
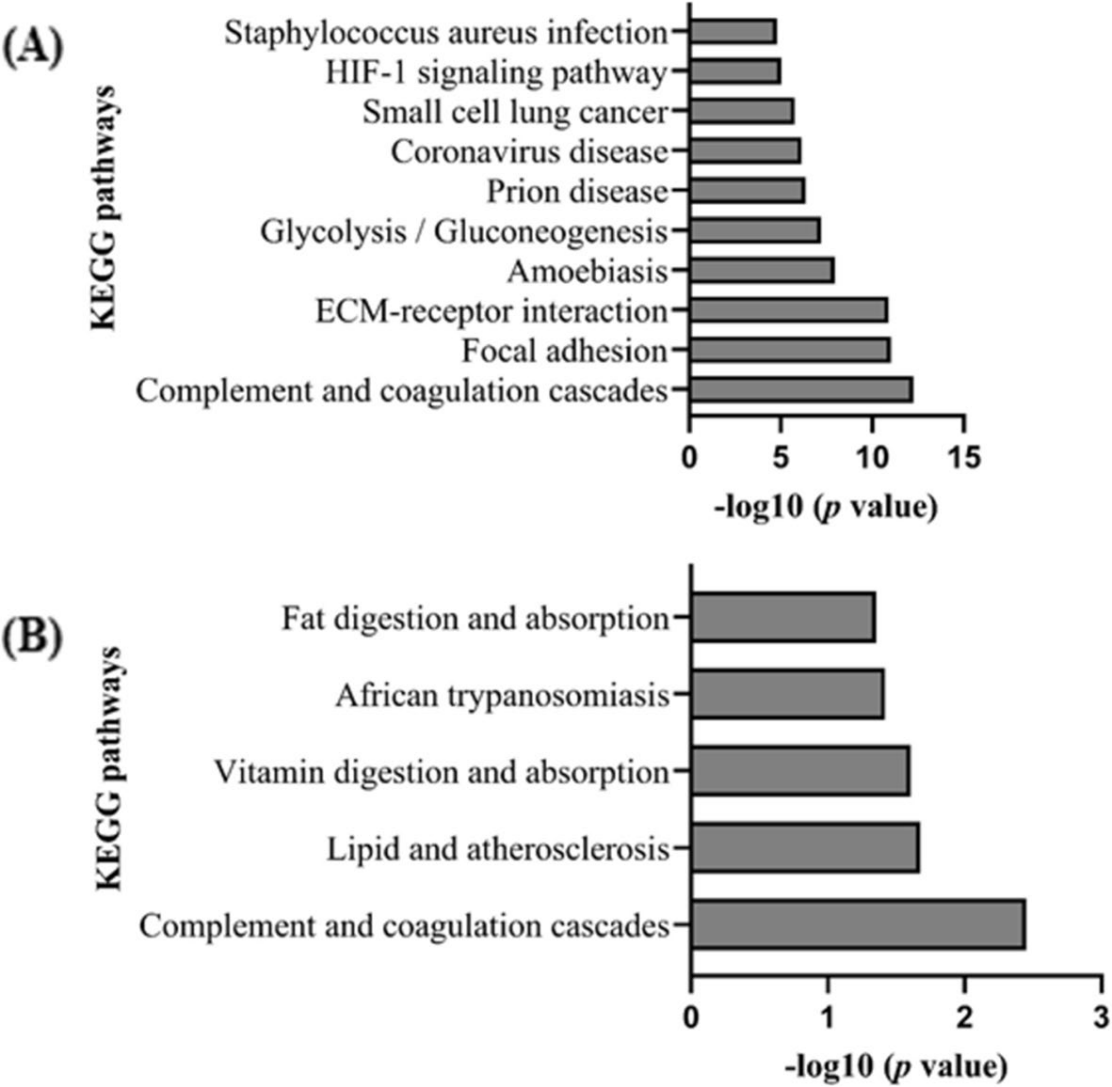
Extracted KEGG pathways of differentially expressed proteins identified by discovery proteomics. **A** Top 10 significantly extracted KEGG pathways of filtered significant proteins derived from aortic tissue discovery phase (T vs CT) using Enrichr. **B** The significantly extracted KEGG pathways derived from the plasma discovery phase (BB vs HC) using Enrichr. KEGG, Kyoto Encyclopedia of Genes and Genomes; T, thoracic aneurysmal tissue sample; CT, control aortic tissue punches; BB, plasma from blood sample collected pre-operatively; HC, healthy control

Pathway enrichment analysis was performed in significantly dysregulated proteins obtained from the discovery phases in both tissue and plasma samples when compared with the controls, in order to reveal possible molecular mechanisms that might be implicated in aneurysm pathology. In total, 89 (see Additional file 3-Table S3.9) and 5 statistically significant enriched Kyoto Encyclopedia of Genes and Genomes (KEGG) pathways were identified in tissue and plasma samples, respectively. The top ten significantly enriched KEGG pathways derived from discovery analysis of aortic tissue samples are shown in Fig. 3A. Specifically, complement and coagulation cascades, focal adhesion and extracellular matrix (ECM)—receptor interaction found to be the top-ranked pathways. The five significantly enriched KEGG pathways derived from the discovery plasma proteomic analysis can be found in Fig. 3B and Additional file 3-Table S3.10. The complement and coagulation cascades were common among the two discovery phases. Irrelevant pathways related with other diseases were not taken into consideration.

**Fig. 4:**
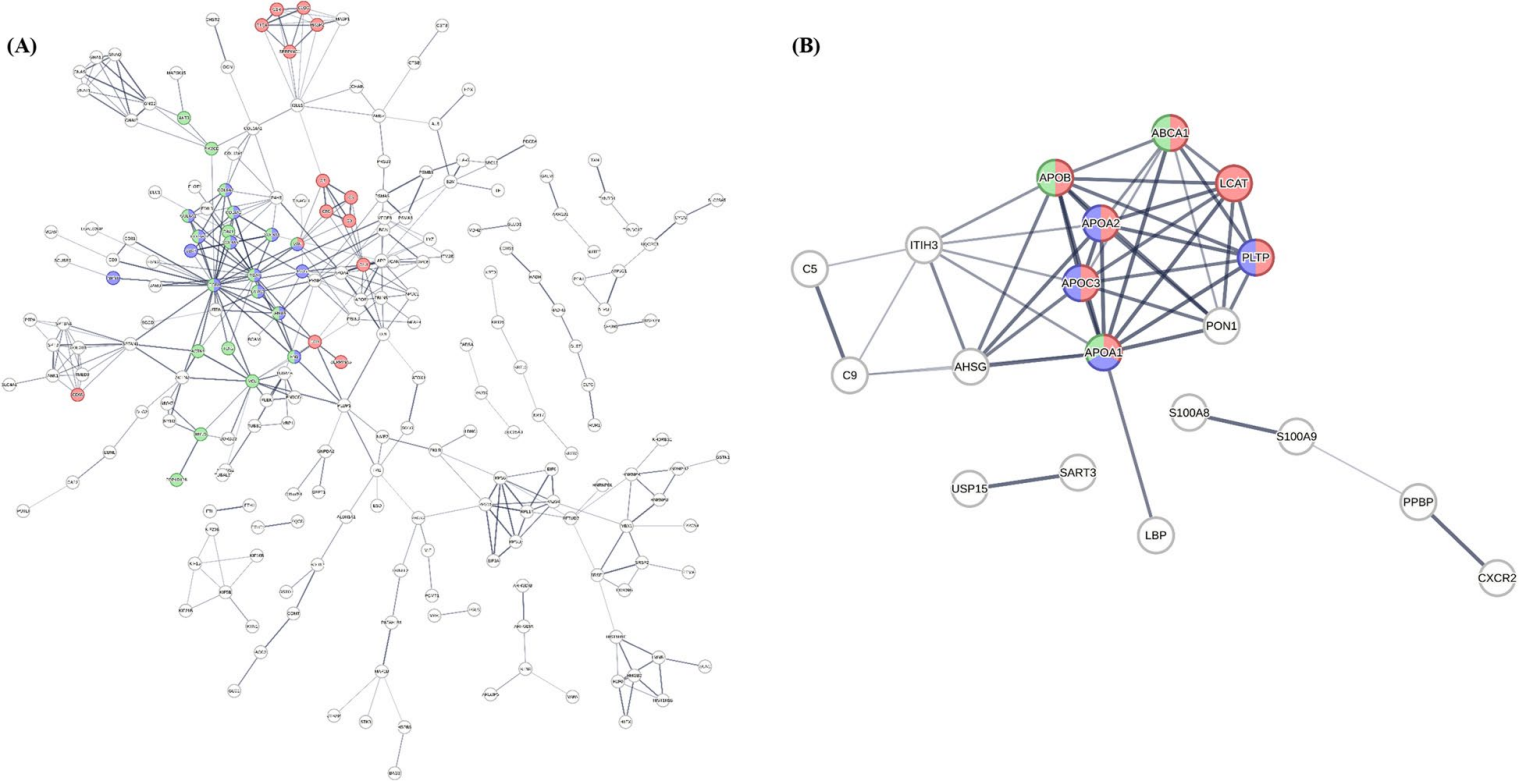
Network analysis to identify the top three pathways derived from the significantly DEPs identified through the aortic tissue and plasma discovery phases. **A** Using STRING database search, we performed pathway analysis using the DEPs with FC ≤ 0.67 or ≥ 1.5 detected in aortic tissue discovery phase (T vs CT). Proteins coloured with red, purple and green are implicated in Complement and Coagulation cascades, ECM-receptor interaction and Focal Adhesion pathways, respectively. White colour nodes represents second shell of interactors. **B** Using STRING database search, we performed pathway analysis using the significantly DEPs with FC ≤ 0.67 or ≥ 1.5 detected in plasma discovery phase (BB vs HC). White colour nodes represents second shell of interactors. Proteins coloured with red, purple and green are implicated in Cholesterol metabolism, PPAR signalling pathway and Fat digestion and absorption, respectively. STRING, Search Tool for Recurring Instances of Neighbouring Genes; ECM, extracellular matrix; BB, plasma from blood sample collected pre-operatively; HC, healthy control

Then, we performed a complementary pathway analysis on the 339 and 18 DEPs identified from discovery aortic tissue and plasma discovery datasets (BB *vs* HC), respectively using STRING database search. The purpose was to obtain a better understanding of the predicted or known interactions among the differentially expressed proteins. The top three significantly enriched KEGG pathways observed after the analysis of 339 DEPs from aortic tissue discovery dataset, includes Complement and Coagulation Cascade (red dots), ECM-receptor interaction (purple dots) and Focal adhesion (green dots), which were also in selected with the Enrichr analysis (Fig. 4A). Due to the lower number of DEPs detected in the plasma discovery phase, a number of interactors were added in order to enrich the network. The top three significantly enriched KEGG pathways derived after STRING analysis includes the Cholesterol metabolism (red dots), PPAR signalling pathway (purple dots) and Fat digestion and absorption (green dots) (Fig. 4B).

### Selection of candidate biomarkers

Since the ultimate goal of the present study was to identify blood-borne TAA biomarkers to distinguish TAA patients and HC, focus was given on DEPs derived from the plasma discovery phase analysis when comparing BB *vs* HC. The significantly DEPs derived from the plasma discovery phase analysis between BB *vs* HC can be found in Additional file 3-Table S3.11. From a total of 18 proteins, only eight proteins followed a specific pattern of expression, such as lower protein expression in BB samples compared to HC samples or vice versa. Specifically, four proteins namely, Kinesin family member 16B (KIF16B), Squamous cell carcinoma antigen recognized by T-cells 3 (SART3), Zinc finger HIT domain-containing protein 2 (ZNHIT2) and Rho guanine nucleotide exchange factor 5 (ARHGEF5) showed the highest expression in BB samples and the lowest expression in the HC samples. Paraoxonase 1 (PON1), Alpha-2-HS-glycoprotein (AHSG), Cyclic nucleotide-gated cation channel alpha-4 (CNGA4) and Dual oxidase 1 (DUOX1) showed the highest expression in the HC samples and the lowest expression in the BB samples. To limit the number of candidate proteins, additional filtering criteria were applied. Only proteins that presented statistically significant differences between affected and control samples in the discovery phase and those that followed a pattern of expression (BB < BA < HC or HC < BA < BB) with a *p-value* ≤ *0.01*, were considered. AHSG was the only protein that remained for further verification and validation analysis.

### Phase 2—Verification of AHSG protein

**Fig. 5:**
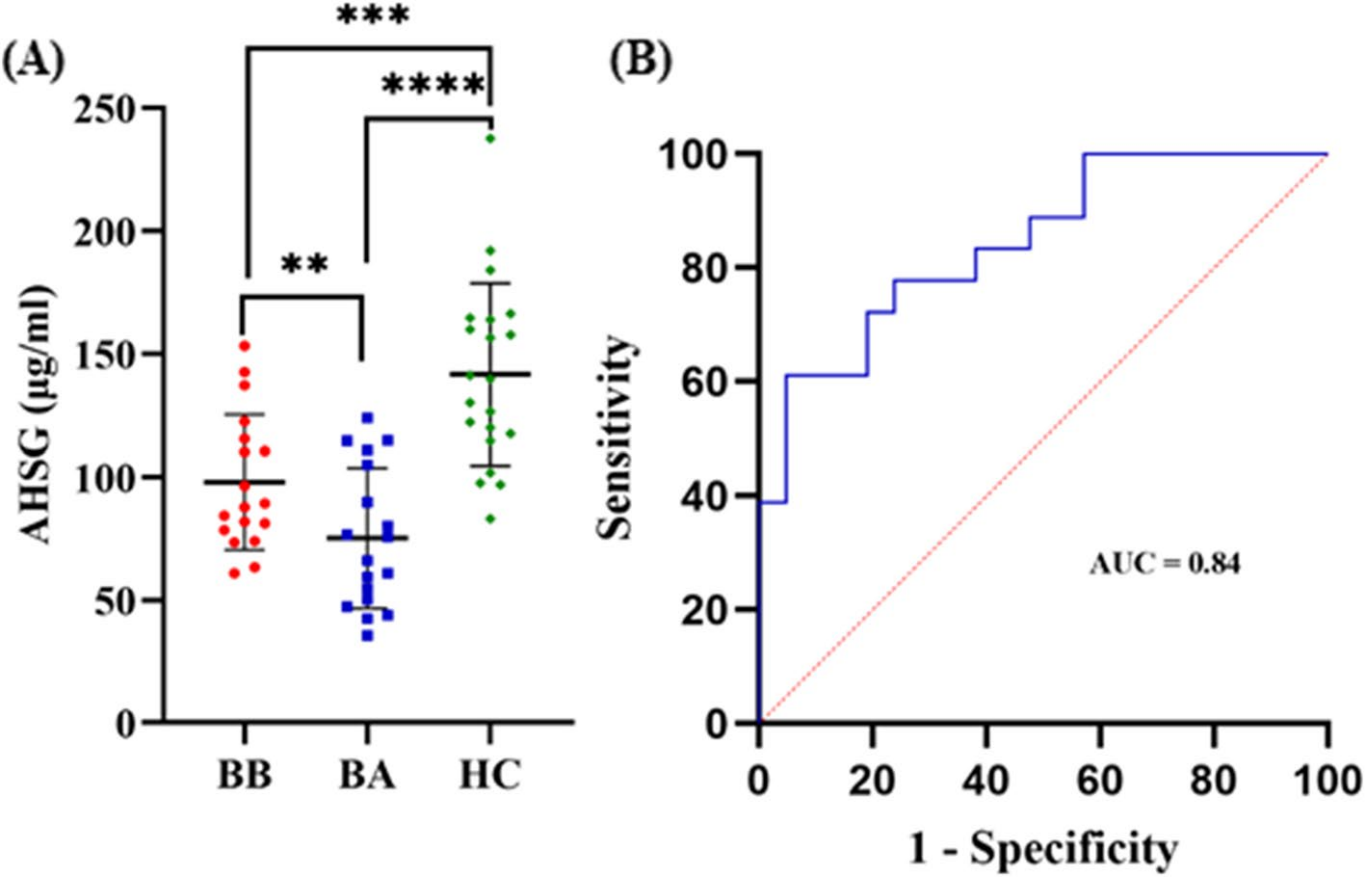
Verification of AHSG protein using targeted LC-MRM-MS.(A) Absolute quantification of AHSG concentration levels in plasma samples from 18 BB, 18 BA and 21 HC. Asterisks indicate the level of statistical significance: * *p* ≤ 0.05, ** *p* ≤ 0.01, *** *p* ≤ 0.001, **** *p* ≤ 0.0001. (B) ROC curve for detection of diagnostic efficiency of AHSG in plasma samples with AUC of 0.84, 95% CI (0.72 to 0.96). BB, plasma from blood sample collected pre-operatively; BA, plasma from blood sample collected 8–10 days post-operatively; HC, healthy controls; AUC, area under the ROC curve

The differential expression levels of AHSG was further explored in 18 BB, 18 BA and 21 HC samples using LC-MRM-MS proteomic analysis. The mean ± SD of AHSG concentrations were determined as 97.9 ± 27, 75.1 ± 29, 141.5 ± 37.1 μg/ml after the conversion from fmol/ul to μg/ml in BB, BA and HC, respectively. Absolute quantification of plasma AHSG showed significantly decreased concentration levels in BB and BA samples compared with the HC (BB *vs* HC, *p* = 0.0002; BA *vs* HC, *p* = 3.46 × 10–^7^). There was also a significant difference (*p* = 0.002) observed between the BB and BA samples (Fig. 5A). The Area Under the Curve (AUC) for AHSG was found to be 0.84 with 95% Confidence Interval (CI) 0.72–0.96 (Fig. 5B).

### Phase 3 – Validation of AHSG protein

**Fig. 6:**
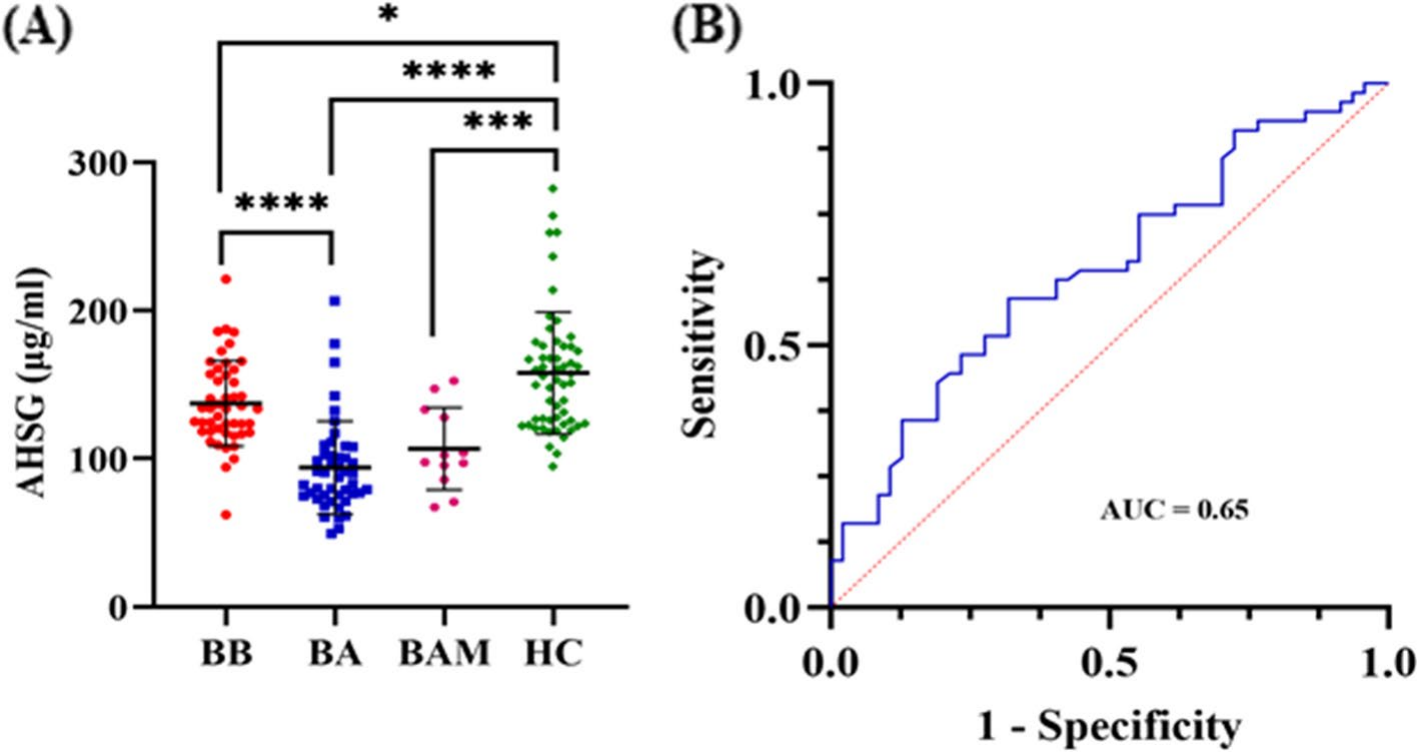
Validation of AHSG protein using ELISA. **A** Quantification of AHSG concentration levels in plasma samples from TAA patients collected at three different time-points (BB, BA, and BAM) compared to HC. Asterisks indicate level of statistical significance: * *p* ≤ 0.05, ** *p* ≤ 0.01, *** *p* ≤ 0.001, **** *p* ≤ 0.0001. **B** ROC curve for detection of the diagnostic efficiency of AHSG in plasma samples with AUC of 0.65, 95% CI (0.54 to 0.75). BB, plasma from blood sample collected pre-operatively; BA, plasma from blood sample collected 8–10 days post-operatively; BAM, plasma from blood sample collected one-month post-operatively; HC, healthy controls; AUC, Area Under the ROC Curve; AHSG, Alpha-2-HS-glycoprotein; TAA, thoracic aortic aneurysm; CI, Confidence Interval

In order to validate the selected protein, we proceeded with the determination of the protein levels of AHSG in a larger number of samples. In total, 47 BB, 46 BA, 12 BAM and 56 HC plasma samples were analysed. A downward trend of the AHSG protein was detected from BB to BA, followed by an upward trend in the BAM samples. The highest AHSG mean concentration was found in the HC samples. Specifically, the mean ± SD of AHSG concentrations were determined as 137 ± 29, 94 ± 31, 107 ± 28, 158 ± 41 μg/ml in plasma samples of TAA patients (47 BB, 46 BA, and 12 BAM) and 56 HC, respectively. (Fig. 6A). Increased levels of the AHSG remained significantly associated with decreased risk of the disease before, after and a month after the operation, as shown by logistic regression analysis adjusted by age; BB *vs* HC, *p* = 0.03; BA *vs* HC, *p* = 3.28 × 10^–7^ and BAM *vs* HC, *p* = 0.00146. The AUC for AHSG levels was found to be 0.65 with 95% Confidence Interval (CI) 0.54–0.75, when comparing BB with HC group (Fig. 6B). Additionally, in this validation set, logistic regression analysis was performed aiming to investigate the association among the classical TAA risk factors. Four risk factors, male sex (OR; 95% CI, 2.60; 1.14–6.17), increasing age (OR; 95% CI, 1.06; 1.01–1.11), hypertension (OR; 95% CI, 11.13; 4.61–29) and hyperlipidaemia (OR; 95% CI, 3.43; 1.50–8.15) were significantly associated with increased TAA risk.

## Discussion

TAA is an indolent disease which develops and progresses asymptomatically with most patients being diagnosed accidentally or after aortic dissection and/or rupture [3]. Risk factors associated with the occurrence of TAA include male sex, advanced age, presence of bicuspid aortic valve, history of smoking, high blood pressure, and hyperlipidaemia [4, 5]. In the present study, analysis of the validation dataset confirmed that male sex, advanced age, hypertension and hyperlipidaemia are associated risk factors for TAAs.

Although there has been significant progress towards diagnosis and therapeutic strategies over the last decade, mortality and morbidity for acute aortic syndrome remains high. Acute aortic syndrome is a variety of acute painful and potentially life-threatening aortic pathologies, including classic acute aortic dissection, intramural haematoma and penetrating atherosclerotic aortic ulcer which shares common clinical characteristics [26]. To reduce the diagnostic time delay which can sometimes be challenging, given the potential for overlapping symptoms attributed to different aetiology, there is an urgent need to improve diagnostic strategies and aim for better outcomes. There is an increased interest in identifying blood-borne biomarkers that can be used in the early diagnosis of TAAs [12]. The application of high-throughput proteomic approaches has paved the way for the discovery of new potential protein biomarkers [27].

This study aimed to identify potential TAA protein biomarkers that could be further pursued with respect to this challenging disease. A proteomic analysis was performed on aortic tissue specimens and plasma samples collected at three different time points (BB, BA and BAM). The tissue and plasma samples were included to reflect the aneurysmal origin of the proteins and their subsequent detection in plasma.

The proteomic analysis led to the identification of significantly DEPs in tissue and plasma samples separately, which were subsequently examined for pathway enrichment analysis. The complement and coagulation cascades were observed as the highest-ranked pathway in the tissue and plasma discovery phases. Both cascades have been considered as individual multicomponent protein networks, with several cross-linkages that have already been found to be associated with inflammation and thrombotic conditions [28]. Previous studies have revealed that complement molecules induce the activation of inflammatory pathways which are implicated in aortic aneurysm formation [29–32]. The ECM-receptor interaction, lipid and atherosclerosis and focal adhesion pathways were also revealed after pathway enrichment analysis. Defects in the components of those pathways [Collagen Type IV Alpha 2 Chain (COL4A2), Collagen Type IV Alpha 1 Chain (COL4A1), Collagen Type VI Alpha 1 Chain (COL6A), Elastin (ELN)] were previously proposed to be associated with aortic aneurysm formation [33]. Then, by performing the complementary pathway analysis using STRING database search, from the plasma discovery phase additional pathways were revealed, e.g. cholesterol metabolism and PPAR signalling pathway. The APOA1 protein was implicated in the top three pathways detected after the analysis of proteins in the plasma discovery phase, showing that there is an interaction with AHSG, suggesting that may be implicated in aneurysm formation. A recently published study showed that the APOA1 oxidation is linked with the dysfunction in high-density lipoproteins in human abdominal aortic aneurysm [34]. Regarding the pathway analysis using STRING database search on the DEPs from tissue discovery phase the top three pathways, Complement and coagulation cascades, ECM-receptor interaction and Focal Adhesion, are consistent with those derived from Enrichr analysis. Vitronectin (VTN) glycoprotein is implicated in the three pathways and may be a promising protein for further investigation in order to reveal any potential association with TAA. Although, there is a plethora of studies implicating various pathways in aneurysm formation, further studies are needed to decipher and understand the complex pathophysiology of TAAs and in particular the interplay of these aforementioned pathways.

The association of AHSG with aortic aneurysm was first examined by Szeberin et *al*. The lower levels of AHSG were detected in sera samples from atherosclerotic aortic aneurysm group and peripheral artery disease patients compared to control group and Marfan disease [35]. A newly published study characterised the insulin-like growth factor binding protein -2 (IGFBP2) as a potential biomarker associated with the thoracic aortic diameter and disease severity, especially in female TAA patients [36]. We found that IGFBP2 was also detected in the discovery phase. This indicates a level of concordance of other datasets, hence the elimination of population specific biases.

The present study resulted in one significant candidate biomarker, AHSG, which differentiated TAA patients from HC individuals. AHSG constitutes a glycoprotein which is mainly synthesized in hepatocytes and secreted in the blood stream, where it interacts with calcium and phosphorus [38]. It is characterized as a multifunctional protein which is involved in processes such as bone development, cell growth and adhesion as well as prevention of ectopic mineralization or hydroxyapatite formation [37–39].

AHSG has been linked to several diseases such as the induction of metabolic dysfunction, insulin resistance, atherosclerosis, inflammation, diabetes mellitus and increased cardiovascular mortality [40–43]. The concentration of circulating AHSG has been shown to predict the risk of vascular calcified plaque, inflammation and all-cause and CVD mortality [44]. A study performed on patients with at least one cardiovascular risk factor or established CVD concluded that patients with high plasma AHSG levels had a lower atherosclerotic plaque progression compared to patients with low AHSG levels [45]. AHSG was additionally proposed as a potential biomarker for the differential diagnosis of ischemic cardiomyopathy from dilated cardiomyopathy, where low levels of AHSG were observed in patients with ischemic cardiomyopathy [46]. It has also been demonstrated that genomic variations in the AHSG gene affect its circulating levels. The non-synonymous polymorphism Thr256Ser was found to be associated with lower AHSG circulating levels and higher mortality rate and inflammation in end-stage renal disease patients [42]. However, other studies did not confirm whether genetic variations are associated with AHSG levels and a CVD risk [47, 48]. To date no genetic variant has been reported for the AHSG gene in TAA in the widely used databases (search performed on 15/12/2022), namely VarSome, Aneurysm Gene Database and ClinVar [49–51]. Further conflicting data regarding the association of AHSG and the pathogenesis of a wide range of vascular disorders have been demonstrated [52–54]. High AHSG levels have been strongly associated with risk of myocardial infarction, ischaemic stroke in general population and early marker of vascular damage in subjects with non-alcoholic fatty liver disease [52, 55]. Also, in patients with coronary artery disease, high concentrations of AHSG were associated with the metabolic syndrome and atherogenic lipid profile [56]. Larger prospective studies are needed to explore the relationship of increased AHSG levels with CVDs.

For the verification and validation of the AHSG in this current study, with respect to ascending TAAs, emphasis was placed on the BB *vs* HC plasma samples. In the two phases, the intermediate BA sample was evaluated to capture any trends as the condition progresses from disease to a normal state. The expression of AHSG in the BA and BAM samples was lower compared to the BB and higher in HC samples. This may be attributed to the short time interval following surgery, thus the levels not reach those for the HC samples. In the validation analysis, an upward trend of the AHSG protein was detected from BA to BAM samples, approaching the levels of the HC samples. This would justify an additional plasma sample from the TAA patients to be collected at six-months post-operatively, which will represent a more comparable near-normal-state condition post-operatively than BAM samples, for the assessment of AHSG. The lower expression of AHSG protein in BB samples compared to HC samples, suggests the potential role of AHSG in aneurysm formation. To the best of our knowledge, this is the first study to investigate the relationship of AHSG in patients with ascending TAA. Thus, further studies are needed to elucidate the role of AHSG protein in TAA pathogenicity.

The present study has some limitations that need to be mentioned. One limitation is the small number of BAM samples. This was due to the difficulty in contacting the patients following their discharge from the AMC as they continue their further medical follow-ups with their personal cardiologists. In Phase 1 of our work, we did not have age-matched samples. The reason was that we included control samples from younger healthy individuals who were free of hypertension, low cholesterol and free of other risk factors in order to increase the power of our discovery stage. Recently a study, showed that aging may play a crucial role in the transformation of human aortic proteome both quantitatively and qualitatively from the healthy status to TAA [57]. Future proteomic studies should include age matched controls to overcome this age difference, following the important findings by Tyrrell et al. 2022. The AHSG, the potential biomarker identified, needs to be tested in a larger cohort of patients and control samples to determine its diagnostic potential. Moreover, no measures of vascular calcification were available in our study in order to speculate potential mechanisms that link lower AHSG levels to higher risk of TAAs.

## Conclusions

This study constitutes a MS-based proteomic investigation for biomarker discovery related to TAAs. Among identified proteins with differential levels between plasma samples collected pre-surgically and control samples, it is suggested that AHSG may be a candidate biomarker for TAA. Plasma AHSG concentrations distinguish TAA patients from HCs, therefore this is a potential blood-borne biomarker for early diagnostic purposes. These results are considered as preliminary and further research is needed to provide insights into the role of the AHSG protein in TAA pathogenesis.

## Supplementary Information

**Additional file 1.** 

**Additional file 2.** 

**Additional file 3.**

## Data Availability

The datasets used and/or analysed during the current study are available in the additional material.

## Abbreviations

AHSG: Alpha-2-HS-glycoprotein
AMC: American Medical Centre
ANOVA: Analysis of Variance
ARHGEF5: Rho guanine nucleotide exchange factor 5
AUC: Area Under the Curve
BA: Blood After
BAM: Blood After One Month
BB: Blood Before
CABG: Coronary Artery Bypass Graft
CI: Confidence Interval
CING: Cyprus Institute of Neurology and Genetics
CNGA4: Cyclic nucleotide-gated cation channel alpha-4
COL4A1: Collagen type IV Alpha 1 Chain
COL4A2: Collagen Type IV Alpha 2 Chain
COL6A: Collagen Type VI Alpha 1 Chain
CT: Control Tissue
CVD: Cardiovascular Disease
DEPs: Differentially Expressed Proteins
DUOX1: Dual Oxidase 1
ECM: Extracellular Matrix
ELISA: Enzyme-Linked immunosorbent Assay
ELN: Elastin
FASP: Filter Aided Sample Preparation
FC: Fold Change
FDR: False Discovery Rate
HC: Healthy Control
IGFBP2: Insulin-like growth factor binding protein 2
KEGG: Kyoto Encyclopedia of Genes and Genomes
KIF16B: Kinesin Family Member 16B
LC-MRM-MS: Liquid Chromatography-Multiple Reaction Monitoring-Mass Spectrometry
LC–MS-MS: Liquid Chromatography-Tandem Mass Spectrometry
MMPs: Matrix Metalloproteinases
MS: Mass Spectrometry
NA: Not available
PC: Principal Component
PCA: Principal Component Analysis
PON1: Paraoxonase 1
ROC: Receiver Operating Characteristic curve
SART3: Squamous cell carcinoma antigen recognized by T-cells 3
SD: Standard Deviation
SIL: Stable Internal Standard
SMCs: Smooth Muscle Cells
STRING: Search Tool for Recurring Instances of Neighbouring Genes
T: Thoracic aneurysmal tissue sample
TAA: Thoracic Aortic Aneurysm
TAA/D: Thoracic Aortic Aneurysm/ Dissection
ZNHIT2: Zinc finger HIT domain- containing protein 2
