# Supplementary material for: Tissue and plasma proteomic profiling indicates AHSG as a potential biomarker for ascending thoracic aortic aneurysms
Source: BMC Cardiovasc Disord. 2023 Mar 16;23:138. doi: 10.1186/s12872-023-03154-6 (PMC10018995; doi:10.1186/s12872-023-03154-6)
Supplement: Supplementary file 2 — Additional file 2. [file 12872_2023_3154_MOESM2_ESM.docx]

| **Accession Number** | **Gene name** | **Peptide Sequence** | **MW**  **(Da)** | **Precursor m/z**  **(Da)** | **Products m/z**  **(Da)** | **Elution times (sec)** | **Collision Energy (eV)** | **Cone Voltage**  **(V)** |
| --- | --- | --- | --- | --- | --- | --- | --- | --- |
| **TARGETED PEPTIDE** | | | | | | | | |
| P02765 | *AHSG* | FSVVYAK | 813.0 | 407.2 | 579.3 [y5 ++] | 6.95 | 17 | 26 |
|  |  |  |  |  | 666.3 [y6 ++] |  |  |  |
|  |  | FSVVYA**K*** | 821.0 | 411.2 | 587.3 [y5 ++] | 6.95 | 17 | 26 |
|  |  |  |  |  | 674.3 [y6 ++] |  |  |  |
| **IMMUNODEPLETED PEPTIDES** | | | | | | | | |
| P02768 | ALB | FQNALLVR | 960.2 | 480.8 ++ | 276.1 [b2+] | 7.85 | 15 | 35 |
|  |  |  |  |  | 500.4 [y4+] |  |  |  |
|  |  |  |  |  | 685.4 [y6+] |  |  |  |
|  |  | FQNALLV**R*** | 970.2 | 485.8 ++ | 276.1 [b2+] | 7.85 | 15 | 35 |
|  |  |  |  |  | 510.4 [y4+] |  |  |  |
|  |  |  |  |  | 695.4 [y6+] |  |  |  |
| P02787 | TF | DGAGDVAFVK | 978.1 | 489.7 ++ | 244.1 [b3+] | 7.9 | 17 | 35 |
|  |  |  |  |  | 464.3 [y4+] |  |  |  |
|  |  |  |  |  | 735.4 [y7+] |  |  |  |
|  |  | DGAGDVAFV**K*** | 986.1 | 493.7 ++ | 244.1 [b3+] | 7.9 | 17 | 35 |
|  |  |  |  |  | 472.3 [y4+] |  |  |  |
|  |  |  |  |  | 743.4 [y7+] |  |  |  |
| P01009 | SERPINA1 | SVLGQLGITK | 1015.2 | 508.3 ++ | 187.1 [b2+] | 9.1 | 16 | 35 |
|  |  |  |  |  | 415.3 [y8++] |  |  |  |
|  |  |  |  |  | 829.5 [y8+] |  |  |  |
|  |  | SVLGQLGIT**K*** | 1023.2 | 512.3 ++ | 187.1 [b2+] | 9.1 | 16 | 35 |
|  |  |  |  |  | 419.3 [y8++] |  |  |  |
|  |  |  |  |  | 837.5 [y8+] |  |  |  |
| P00738 | HP | TEGDGVYTLNNEK | 1447.5 | 720.3 ++ | 881.4 [y7+] | 6.6 | 26 | 35 |
|  |  |  |  |  | 1037.5 [y9+] |  |  |  |
|  |  |  |  |  | 1209.6 [y11+] |  |  |  |
|  |  | TEGDGVYTLNNE**K*** | 1455.5 | 724.3 ++ | 889.4 [y7+] | 6.6 | 26 | 35 |
|  |  |  |  |  | 1045.5 [y9+] |  |  |  |
|  |  |  |  |  | 1217.6 [y11+] |  |  |  |

**Table S2.1: Parameters for the synthesized peptides of targeted and immunodepleted proteins employed during LC-MRM-MS**
